# *Plasmodium falciparum *transmission and aridity: a Kenyan experience from the dry lands of Baringo and its implications for *Anopheles arabiensis *control

**DOI:** 10.1186/1475-2875-10-121

**Published:** 2011-05-14

**Authors:** Albert O Mala, Lucy W Irungu, Josephat I Shililu, Ephantus J Muturi, Charles M Mbogo, Joseph K Njagi, Wolfgang R Mukabana, John I Githure

**Affiliations:** 1 Human Health Division, International Centre of Insect Physiology and Ecology, P.O. Box 30772-00100, Nairobi, Kenya; 2 School of Biological Sciences, University of Nairobi, Nairobi, Kenya; 3 Illinois Natural History Survey, University of Illinois at Urbana-Champaign, Illinois, USA; 4 Kenya Medical Research Institute, Kenya; 5 Ministry of Health, Division of Malaria Control, Nairobi, Kenya

## Abstract

**Background:**

The ecology of malaria vectors particularly in semi-arid areas of Africa is poorly understood. Accurate knowledge on this subject will boost current efforts to reduce the burden of malaria in sub-Saharan Africa. The objective of this study was to describe the dynamics of malaria transmission in two model semi-arid sites (Kamarimar and Tirion) in Baringo in Kenya.

**Methods:**

Adult mosquitoes were collected indoors by pyrethrum spray collections (PSC) and outdoors by Centers for Disease Control (CDC) light traps and identified to species by morphological characteristics. Sibling species of *Anopheles gambiae *complex were further characterized by rDNA. PCR and enzyme-linked immuno-sorbent assays (ELISA) were used to test for *Plasmodium falciparum *circumsporozoite proteins and host blood meal sources respectively.

**Results:**

*Anopheles arabiensis *was not only the most dominant mosquito species in both study sites but also the only sibling species of *An. gambiae s.l. *present in the area. Other species identified in the study area were *Anopheles funestus*, *Anopheles pharoensis *and *Anopheles coustani*. For Kamarimar but not Tirion, the human blood index (HBI) for light trap samples was significantly higher than for PSC samples (Kamarimar, 0.63 and 0.11, Tirion, 0.48 and 0.43). The HBI for light trap samples was significantly higher in Kamarimar than in Tirion while that of PSC samples was significantly higher in Tirion than in Kamarimar. Entomological inoculation rates (EIR) were only detected for one month in Kamarimar and 3 months in Tirion. The number of houses in a homestead, number of people sleeping in the house, quality of the house, presence or absence of domestic animals, and distance to the animal shelter and the nearest larval habitat were significant predictors of *An. arabiensis *occurrence.

**Conclusion:**

Malaria transmission in the study area is seasonal with *An. arabiensis *as the dominant vector. The fact this species feeds readily on humans and domestic animals suggest that zooprophylaxis may be a plausible malaria control strategy in semi-arid areas of Africa. The results also suggest that certain household characteristics may increase the risk of malaria transmission.

## Background

The main malaria vectors in sub-Saharan Africa (*Anopheles gambiae*, *Anopheles arabiensis *and *Anopheles funestus*) are confronted with highly variable and challenging climatic conditions especially in the semi arid regions and during the dry season [1]. A common challenge in these areas is the sudden shrinking or complete disappearance of the larval habitats during the dry season. This loss in larval habitats is followed by a rapid drop in abundance of malaria vectors and concomitant decrease in the incidence of severe malaria [2,3]. However the onset of the rainy season results in surprisingly rapid increase in malaria vector populations [4-6]. It is unclear whether the initial population buildup results from a new founder population of immigrants from neighboring areas with more permanent larval habitats or expansion from a very small local population that survives the dry period. Previous ecological studies suggest that *Anopheles *eggs have low tolerance to desiccation implying that only adults can survive the dry season [7]. These adults may hide in shelters such as rodent burrows, abandoned houses and wells, minimizing the chances of their detection through pyrethrum spray collections [8]. Further, despite local reduction in density during the dry season, population genetic studies suggest that *An. arabiensis *which is the most common vector in semi-arid areas, maintains large permanent deme over a large area [9].

In addition to vector density, variations in environmental conditions have the potential to influence other factors that are directly related to malaria transmission. Environmental factors experienced by immature stages often influence the size of subsequent adults, which in turn determines the probability of adult survival. Further, other factors such as host availability and accessibility determine the likelihood of a mosquito vector to acquire and transmit malaria parasites. *Anopheles arabiensis *show plastic responses in host feeding patterns and readily diverts to feeding on the most common and easily accessible host(s) [10,11]. For this reason, zooprophylaxis is considered a practical strategy for reducing malaria transmission by this species [12].

Entomological inoculation rate (EIR), the average number of infective bites per person per unit time is the standard index used to estimate the intensity of malaria transmission in a given area. EIR is computed as the product of the mosquito human-biting rate and the proportion of mosquitoes carrying sporozoites (sporozoite rate) in their salivary glands [13]. Exceedingly low sporozoite rates (SR) have continued to hinder accurate determination of this critical entomologic index in Africa, especially in areas of unstable transmission where EIR is often below the undetectable levels [14,15]. The objective of this study was to examine the seasonal changes in malaria vector populations and the intensity of malaria transmission in two model semi-arid villages in Kenya. Knowledge generated from this study will guide the development of effective malaria control strategies in semi-arid areas of Africa.

## Methods

### Ethical considerations

Permission to carry out this study was obtained from the Ministry of Health, and ethical clearance was properly sought from the National Ethical Review Committee of Kenya but was deemed unnecessary. A sensitization rally was organized with the population during which the purpose of the study was clearly explained. Verbal consents from household heads of the study houses used in this research work were obtained only after informing them of the rationale and methodology of the research work.

### Study area

**Figure 1:**
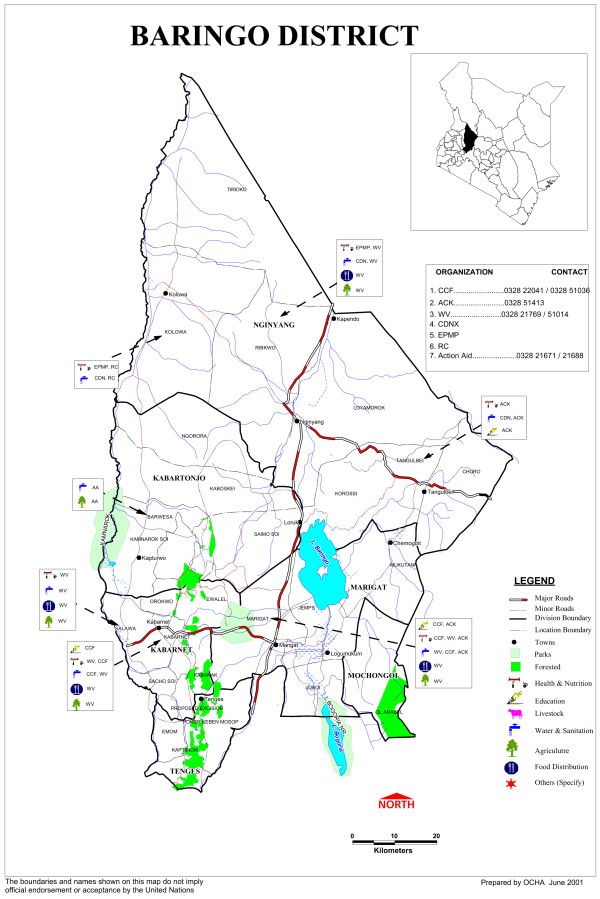
**Map of Baringo district**.

The study was based in Marigat division of Baringo district, Kenya. Marigat town is approximately 250 km north-west of Nairobi. The town is situated 0.45 N, 36 E and about 700 meters above the sea-level, most of which is rangelands (Figure 1). The study area is characterized by high temperatures (above 32°C) and low rainfall (500-600 mm) conditions that lead to rapid loss of transient larval habitats. The rainy season occurs from April to July while the peak dry season commences in November and ends in February of every year. Two study sites; Kamarimar and Tirion were selected for this study based on their proximity to the permanent Loboi swamp. The former is adjacent to the swamp and the latter is approximately 3 km away from the swamp. The study sites are described in details elsewhere [16].

### Mosquito sampling and identification

**Figure 2:**
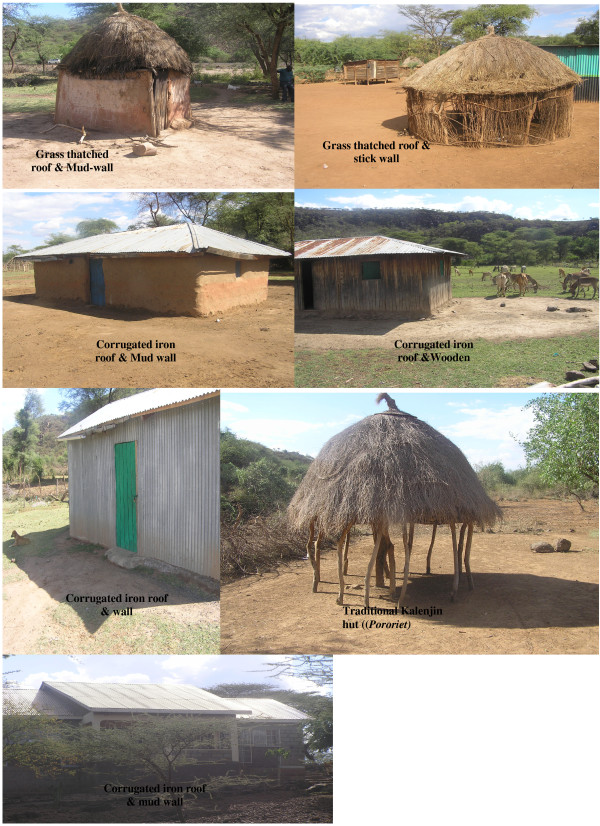
**House types two rural villages in a semi arid area in Baringo,, Kenya**.

In each study site, mosquitoes were collected in 10 randomly selected houses by pyrethrum spray collections (PSC). The collections were done once every week between 0700 and 1100 hours for a period of 22 months (July 2008 - April 2010). The houses were categorized into six types depending on materials used to construct the walls and the roofs (Figure 2). Other household characteristics that were recorded include size of eaves, distance to the nearest animal shed, use of insecticide treated bed nets and distance to the nearest habitat. Every week, a Centers for Disease Control (CDC) and prevention light trap (J.W. Hock Ltd, Gainesville, FL, U.S.A.) was also operated in the main bedroom of each of the 10 houses between 18.00 and 06.00 hours to estimate the human biting rates. To sample outdoor mosquito populations, 10 ten CDC light traps were operated outdoor during the night preceding PSC collection once weekly. The samples were transported to the laboratory and female *Anopheles *were identified to species using morphological characteristics [17,18]. The samples were scored as unfed, blood-fed, semi-gravid or gravid by examining their abdomen under a dissecting microscope and later preserved in labeled vials containing anhydrous calcium sulphate. Random samples of *An. gambiae *complex were identified to their respective sibling species by rDNA PCR method [19].

### Determination of blood meal source using ELISA-based methods

The abdomen from each blood-fed mosquito was homogenized in 50 μl of phosphate buffered saline (PBS) and 950 μl of grinding buffer. Blood meals were identified by direct enzyme-linked immunosorbent assay (ELISA) anti-host IgG conjugate against human and bovine in a single-step assay. Any non-reacting samples were subsequently tested using goat IgG. Fifty microlitres of the mosquito triturate was added to U-shaped bottom 96-well microtitre plates and incubated overnight at room temperature. Each well was then washed twice with PBS containing 0.5% Tween 20, followed by addition of 50 μl of host specific conjugate in 0.5% boiled casein containing 0.025% Tween 20. The ELISA results were read visually according to the protocol of Beier *et al *[20]. The human blood index (HBI) and bovine blood index (BBI) were calculated as the proportion of blood-fed mosquito samples that had fed on either human or bovine out of the total tested.

### ELISA detection of *Plasmodium falciparum *sporozoite infections

The head and thorax portion of each individual mosquito was tested for *P. falciparum *circumsporozoite proteins (CSPs) by ELISA [21]. The sporozoite rate (SR) was computed as the proportion of mosquitoes positive for *P. falciparum *CSPs out of the total tested. EIR was derived as the product of human biting rate (HBR) estimated as the geometric mean number of vectors caught in a light trap/per house and the sporozoite rate.

### Meteorological data

A rain gauge (Tru-Chek^®^, Rain Gauge Division, Edwards Manufacturing Co. Albert Lea, MN, U.S.A.) was placed in each of the study villages and rainfall data recorded daily over a period of 22 months (July 2008 to April 2011). Temperature and relative humidity were measured using temperature and relative humidity data loggers (Onset Computer Corporation, Bourne, MA, U.S.A.).

### Statistical analysis

Data was analysed using Statistical Analysis Software (SAS) Version 9.1 (SAS Institute). Data were checked for normality and homogeneity of variances and analysis of variance (ANOVA) was used to compare the mean differences in adult mosquito densities between villages and months. Chi-square and Fishers exact tests were used (as appropriate) to compare the differences in the human blood index (HBI), SR and HBR of *An. arabiensis *between seasons and villages. Pearson Correlation analysis was used to asses the relationship between rainfall and adult mosquito densities while multivariate logistic regression analysis was used to assess the relationship between the measured micro-epidemiological characteristics and the occurrence of *An. arabiensis*.

## Results

### Meteorology

The total precipitation for the period September 2008 to March 2010 was 100 mm. The rainy season for the year 2008 was concentrated in July-August, bimodal in 2009 concentrated in January-February and September-December. The months of February-March were wet in the year 2010. The average daily temperature was 26.24 (range = 21.60-30.68) and the average relative humidity was 20.94% (range = 2.74-94.8%).

### Species composition and abundance

Four *Anopheles *species were collected in the two study sites during the 22-month period (Table 1). These included *An. gambiae s.l. *(66.8%), *An. funestus *(17.9%), *An. pharoensis *(14.5%) and *An. coustani *(0.8%). *Anopheles gambiae s.l. *and *An. coustani*, respectively, were the most and the least abundant species in both study sites. *Anopheles pharoensis *was second most abundant species in Kamarimar, while *An. funestus *was the second most abundant species in Tirion. For all species, light trap collections were more productive than PSC. rDNA PCR analysis of 500 *An. gambiae *s.l samples revealed *An. arabiensis *as the only sibling species present in the study area.

**Table 1:** Relative abundance of *Anopheles *mosquito species in the two study sites in Baringo, Kenya

Mosquito species	Kamarimar		Tirion	
	Light trap (%)	PSC (%)	Light trap (%)	PSC (%)
*Anopheles funestus*	800 (11.9)	280(22)	1840(24.7)	72(5.8)
*Anopheles gambiae*	4186 (62.2)	799(62.9)	5246(70.4)	907(73.7)
*Anopheles pharoensis*	1700 (25.3)	180(14.2)	300(4.0)	240(19.5)
*Anopheles coustani*	42(0.62	12(0.9)	67(0.9)	12(1.0))

**Overall Total**	**6728**	**1271**	**7453**	**1231**

### Spatial and temporal distribution of mosquitoes

**Figure 3:**
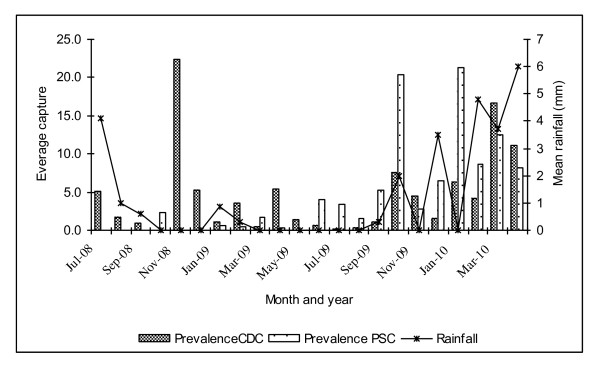
**Month to month variation in pooled mean number of indoor resting mosquitoes from Kamarimar and Tirion relative to rainfall patterns**.

The densities of *An. arabiensis *were significantly higher in Tirion than in Kamarimar (F = 10.76, df = 1,*P *= 0.001) and were mostly collected in CDC light traps (outdoors) than in pyrethrum spray collections (F = 7.58, df = 1, *P *= 0.001). There were significant differences in adult densities across months in both Kamarimar (F = 6.66, df = 18, P = 0.001), and Tirion (F = 6.77, df = 18, P = 0.001). Adult densities were generally low with peaks in the months of July and November 2008, February, April and October 2009. However, there was no clear cut difference in monthly adult densities in the year 2010 (Figure 3). These populations were virtually undetectable in October 2008 and July 2009. The year 2010 experienced a lot of rainfall in the months of March and April, with *An. arabiensis *densities remaining generally high but with low densities being observed in the dry months of January and February. When mosquito densities between wet the months and dry months were compared, no significant differences were observed. Similarly, there was no significant correlation between rainfall and adult mosquito density (r = 0.01, df = 20, P > 0.05).

### Blood meal sources

Blood meal hosts of 417 (95%) samples out of the 421 tested were successfully identified by ELISA (Table 2). In Kamarimar, the human blood index (HBI) including those with mixed blood meals for outdoor collected (CDC light traps) samples was 0.63 and significantly higher than 0.11 for indoor collected (PSC) samples (χ^2 ^= 30.938, P < 0.0001). Similar values for Tirion were 0.48 and 0.43, for outdoor and indoor collected samples respectively and did not differ significantly from each other (χ^2 ^= 0.522, P = 0.478). The HBI for outdoor collected samples was significantly higher in Kamarimar (0.65) than in Tirion (0.48, χ^2 ^= 4.899, P = 0.027). In contrast, the HBI for indoor collected samples was significantly higher in Tirion (0.43) than in Kamarimar (0.11, χ^2 ^= 14.847, P < 0.0001). Mixed blood feeding was also observed, the most common being bovine and goat blood meals. Some samples contained blood meals from all the three hosts tested.

**Table 2:** Blood-meal sources of *Anopheles arabiensis *mosquitoes in the two study sites, Baringo, Kenya.

	Kamarimar		Tirion	
		
	CDC (n; %)	PSC (n; %)	CDC (n; %)	PSC (n; %)
Human	45 (51.1)	0 (0)	56 (37.6)	33 (24.3)
Bovine	0 (0.0)	21 (47.7)	30 (20.1)	18 (13.2)
Goat	0 (0.0)	0 (0.0)	8 (5.4)	4 (2.9)
Human and Bovine	9 (10.2)	0 (0.0)	5 (3.4)	18 (13.2)
Human and Goat	0 (0.0)	0 (0.0)	2 (1.3)	3 (2.2)
Bovine and Goat	33 (37.5)	18 (40.9)	40 (26.8)	55 (40.4)
All	1 (1.1)	5 (11.4)	8 (5.4)	5 (3.7)

Total	88	44	149	136

### Human biting density, sporozite rates and EIR for *Anopheles arabiensis*

The HBR of 9.51 bites per person per month in Kamarimar was not significantly different from 8.93 bites per person per month in Tirion (F = 0.79, df = 1, *P = *0.3812). For each of the three years, there was a marked monthly variation in HBR. HBR was undetectable in some months, and quite high in other months e.g. 96 bite/person for the month of November 2008. Sporozoite rates were very low (16 out of 7,322, 0.02%) and were only detected in two months in Kamarimar (October 2009 and March 2010) and four months in Tirion (October and November 2009 and January and March 2010 (Table 3). Similarly, EIR was undetectable throughout much of the study period except in October 2009 in Kamarimar and October 2009, November 2009 and January 2010 in Tirion when low levels were detected.

**Table 3:** *Anopheles arabiensis *sporozoite rates, Human biting rates and entomological inoculation rates in two villages in Baringo, Kenya

		Kamarimar			Tirion		
**Month**	**Year**	**SR**	**HBR**	**EIR**	**SR**	**HBR**	**EIR**

July	2008	0.0000	46.6226	0.0000	0.0000	0.0000	0.0000
August	2008	0.0000	12.0532	0.0000	0.0000	3.8843	0.0000
September	2008	0.0000	2.9356	0.0000	0.0000	3.1748	0.0000
October	2008	0.0000	0.0000	0.0000	0.0000	0.0000	0.0000
November	2008	0.0000	13.8905	0.0000	0.0000	95.7889	0.0000
December	2008	0.0000	0.0000	0.0000	0.0000	0.0000	0.0000
January	2009	0.0000	0.0000	0.0000	0.0000	0.0000	0.0000
February	2009	0.0000	0.0000	0.0000	0.0000	0.0000	0.0000
March	2009	0.0000	3.1910	0.0000	0.0000	3.5652	0.0000
April	2009	0.0000	4.0000	0.0000	0.0000	0.0000	0.0000
May	2009	0.0000	0.0000	0.0000	0.0000	0.0000	0.0000
June	2009	0.0000	15.6254	0.0000	0.0000	7.1680	0.0000
July	2009	0.0000	6.8903	0.0000	0.0000	7.0405	0.0000
August	2009	0.0000	3.1072	0.0000	0.0000	3.5569	0.0000
September	2009	0.0000	9.7898	0.0000	0.0000	12.4831	0.0000
October	2009	0.0019	62.1170	0.1176	0.0058	22.8941	0.1339
November	2009	0.0000	31.0782	0.0000	0.6667	38.4435	0.0000
December	2009	0.0000	11.2909	0.0000	0.0000	11.0009	0.0000
January	2010	0.0000	32.5275	0.0000	0.0085	33.3302	0.0000
February	2010	0.0000	39.7993	0.0000	0.0000	34.5888	0.0000
March	2010	0.0027	0.0000	0.0000	0.0066	0.0000	0.0000

July	2010	0.0000	0.0000	0.0000	0.0000	0.0000	0.0000

HBR = human biting rate; SR = sporozoite rate; EIR = entomologic inoculation rate. The sporozoite rate was derived from indoor resting collection. EIR = infective bites/person/month.

### Household characteristics and occurrence of *An. arabiensis*

Multivariate logistic regression analysis was used to determine the relationship between the measured household characteristics and occurrence of *An. arabiensis *(Table 4). The odds of *An. arabiensis *occurrence increased with decreasing distance to the animal shelter and the nearest larval habitat and increasing number of houses, sleepers and size of eaves. *An. arabiensis *was also more likely to be encountered in grass-thatched than in metal-roofed houses and in the absence than in presence of animals.

**Table 4:** House characteristics and indoor resting densities of *Anopheles arabiensis *in the two study sites in Baringo, Kenya

Characteristic	Coefficient	S.E.	df	Sig.	Odds ratio
Number of houses	0.16	0.04	1	< 0.0001	1.17
Number of sleepers	0.28	0.07	1	< 0.0001	1.76
Presence of animals	-0.91	0.42	1	0.031	0.40
Distance to animal shelter	-0.13	0.02	1	< 0.0001	0.88
Roofing material	-0.76	0.39	1	0.048	0.47
Size of eaves	0.06	0.01	1	< 0.0001	1.06
Distance to the nearest larval habitat	-0.04	0.00	1	0.003	0.81

## Discussion

*Anopheles arabiensis *was the main vector of malaria in the study area and feeds readily on both humans and domestic animals. Malaria transmission by this species was largely undetectable throughout much the study period except in a few wet months when low levels of transmission were detected. Suggesting that transmission of this disease in arid and semi-arid areas of Africa is mainly seasonal. The finding that *An. arabiensis *is the only sibling species of *An. gambiae *s.l. present in the study area is consistent with previous cytogenetic studies [22-25]. Some *An. funestus *were collected during the study period. However, this species is known to thrive well in hot and humid environments as opposed to the hot and dry conditions [26-29]. It is also possible that lack of suitable long-lasting habitats preferred by this species may partly account for the low densities witnessed in the current study [30]. Previous invesigations in the study area reported production of *An. arabiensis *in Kamarimar is sustained by permanent or semi-permanent larval habitats that included pan dams, marshes and adjoining drainage canals used for irrigation that are less dependent on rainfall [16].

A significant fraction of anthropophagic mosquitoes were sampled outdoors from the two villages, a clear evidence of house-exiting by vectors after feeding on humans. Conversely, large fractions of zoophilic mosquitoes were collected indoors illustrating the potential of employing zooprophylaxis as a strategy as has happened in some parts of Africa, Europe and USA [10]. The role of human activities in increasing human-vector contact cannot, however be ruled out. Lightly-dressed residents in the study villages stay out late in the evening to irrigate their farms before temperatures sky-rocket during day time, exposing themselves to mosquito bites. Herding is a common practice in Baringo and indeed, most semi-arid areas of Africa. The largest communal grazing field in the study areas is located in Tirion and is used by hundreds of pastoralists from different villages. Increasing herd sizes could be a plausible vector control strategy but may be counterproductive under certain circumstances where high livestock densities lead to an increase in vector densities and high human biting rates [31,32].

The intensity of malaria transmission by *An. arabiensis *as measured by EIR was extremely low and seasonal. This could be due to low infectiousness of the human population in the area or because a majority of mosquitoes caught by light traps were newly emerged and had not had an opportunity to acquire an infectious blood meal. Further, it is also likely that adult lifespan of this species was shorter than the extrinsic incubation period for malaria parasites but this possibility was not evaluated in the current study. These findings corroborate those from other semi-arid areas of Africa in Sudan and Eritrea. Shililu and others [33] established that the risk of exposure to sporozoite-laden *An. arabiensis *in Eritrea was highly heterogeneous and seasonal. Biting rates and EIRs peaked during the rainy season, but little or no transmission occurred during the dry season. Year-long studies conducted in the Sudan by Dukeen and Omer [34] in the 1970s showed *An. arabiensis *adult densities peaking seasonally during certain months when Nile water levels were low, and rising when the water levels rose.

Entry and/or exit of mosquitoes largely depended on house type. The average number of indoor resting mosquitoes collected in both villages varied significantly depending on the type of house occupied by potential human hosts, highlighting the importance of this micro-epidemiological factor in malaria transmission. There was a strong preference for grass-thatched houses, making house modification to limit mosquito flight into houses a plausible control strategy. This finding reinforces the common belief that poverty is a major driver of malaria transmission in Africa. Most communities in the continent's rural and resource constrained areas are largely unable to afford decent housing with adequate screening measures to block mosquito entry into houses.

It is encouraging that an EIR value was realized in only one out of the 22 months in which sampling was done. This is a positive outcome in the sense that increasing uptake of malaria prevention measures through integrated vector management programs could lower the current prevailing EIR levels to below one infective bite per person per month. Such programs have the potential of eliminating malaria in these areas by reducing parasite rates to levels that can interrupt malaria transmission. We believe these findings have important implications and will inform policy on vector control in epidemiological zones of the world where low seasonal malaria transmission patterns are experienced.

## Conclusion

Malaria transmission in the study area was seasonal and vectored by *An. arabiensis*. This species was found to feed readily of humans and domestic animals suggesting that zooprophylaxis may be a potential malaria control strategy in the study area and other semi-arid regions of Africa. However, further studies are needed to assess the negative impacts of this strategy in target sites.

## Competing interests

The authors declare that they have no competing interests.

## Authors' contributions

AOM conducted the field studies, analyzed the data and wrote the manuscript. JIS and CMM provided scientific guidance in data collection, analysis and manuscript preparation and planning, and implementation of day-to-day field and laboratory activities. JKN and LWI offered scientific guidance in data analysis and manuscript preparation. EJM and WRM provided scientific guidance in data analysis, interpretation of results and manuscript preparation. JIG provided overall supervision of the study and preparation of manuscript. All authors actively contributed to the interpretation of the findings and development of the final manuscript and approved the final manuscript.
